# Full-Genome Analysis of a Canine Pneumovirus Causing Acute Respiratory Disease in Dogs, Italy

**DOI:** 10.1371/journal.pone.0085220

**Published:** 2014-01-06

**Authors:** Nicola Decaro, Pierfrancesco Pinto, Viviana Mari, Gabriella Elia, Vittorio Larocca, Michele Camero, Valentina Terio, Michele Losurdo, Vito Martella, Canio Buonavoglia

**Affiliations:** Department of Veterinary Medicine, University of Bari, Valenzano, Italy; Virginia Polytechnic Institute and State University, United States of America

## Abstract

An outbreak of canine infectious respiratory disease (CIRD) associated to canine pneumovirus (CnPnV) infection is reported. The outbreak occurred in a shelter of the Apulia region and involved 37 out of 350 dogs that displayed cough and/or nasal discharge with no evidence of fever. The full-genomic characterisation showed that the causative agent (strain Bari/100-12) was closely related to CnPnVs that have been recently isolated in the USA, as well as to murine pneumovirus, which is responsible for respiratory disease in mice. The present study represents a useful contribution to the knowledge of the pathogenic potential of CnPnV and its association with CIRD in dogs. Further studies will elucidate the pathogenicity and epidemiology of this novel pneumovirus, thus addressing the eventual need for specific vaccines.

## Introduction

Canine infectious respiratory disease (CIRD) is a multifactorial disease affecting dogs of all ages, which is typically induced by simultaneous viral and bacterial infections [1]. Apart from well-known canine respiratory pathogens, such as canine adenovirus type 2, canine herpesvirus [2], canine distemper virus [3], and canine parainfluenza virus [4], novel viruses are being continuously associated with CIRD occurrence in dogs. These include canine influenza virus [5], canine respiratory coronavirus [6], canine pantropic coronavirus [7]–[8], canine bocaviruses [9], and canine hepacivirus [10].

Pneumoviruses (family *Paramyxoviridae*, subfamily *Pneumovirinae*, genus *Pneumovirus*) are enveloped, single-strand negative-sense RNA viruses that are associated with respiratory disease in mammals and birds. Apart from the prototype species human respiratory syncytial virus (HRSV) and its ruminant relative bovine respiratory syncytial virus (BRSV), a murine pneumovirus (MPV), also known as pneumonia virus of mice, is included in the genus *Pneumovirus* [11]. This virus, which is only distantly related to human and ruminant RSVs, is a natural rodent pathogen circulating among research and commercial rodent colonies [12].

Recently, a pneumovirus was associated to respiratory disease in canine breeding colonies in the United States [13]–[14]. The virus, designated as canine pneumovirus (CnPnV), was found to be very closely related to MPV, displaying 95% nucleotide identity with the MPV prototype isolate J3666 [14]. Experimental infection of mice with the canine isolate demonstrated that CnPnV is able to replicate in the mouse lung tissue inducing pneumonia [15]. Although the virus was discovered more than 4 years ago, to date there is no complete genomic sequence, which prevents a comprehensive comparative study with other members of the *Pneumovirinae* subfamily.

The aim of the present manuscript is to report the detection and molecular characterisation of this emerging virus in dogs with respiratory disease in Italy. The full-length genome of a prototype strain was determined and analysed in comparison with American strains and other pneumoviruses.

## Materials and Methods

### Ethics Statement

The study did not involve any animal experiment. Only sample collection from naturally infected dogs was carried out, consisting of a single nasal swab per dog. This was needed for the laboratory analyses and did not involve any suffering of the sampled animals.

### Clinical Outbreak and Sample Collection

In 2012, an outbreak of CIRD occurred in a canine shelter of the Apulia region, southern Italy, involving 37 out of 350 housed animals. All the dogs involved were mixed bred and included animals aged from 2.5 months to 12 years; neither age nor gender predisposition was evident for the CIRD occurrence. Pest control was carried out only sporadically in the shelter, but there were no systematic measures against rodent and insect populations. Housed dogs were routinely vaccinated against canine parvovirus (CPV), canine distemper (CDV), canine adenoviruses (CAdVs) and *Leptospira* spp., while no specific vaccine for prophylaxis of CIRD was employed. Respiratory signs were generally mild consisting of cough and/or nasal discharge with no evidence of fever. Haematological parameters were not evaluated. Nasal and pharyngeal swabs were collected from two CIRD-affected mixed-breed dogs, a 7-month-old male and a 3-year-old female. Swabs were immersed in 1.5 ml viral transport medium consisting of Dulbecco’s modified Eagle’s medium (DMEM) supplemented with 5% fetal calf serum (FCS), 1000 IU/ml penicillin, 1000 µg/ml streptomycin and 10 µg/ml amphotericin B.

### RNA Extraction

Aliquots of the nasal and pharyngeal swab extracts were combined and subsequently clarified by centrifuging at 2,500×*g* for 10 min. One-hundred-forty microliters of the supernatants were then used for RNA extraction by means of QIAamp® Viral RNA Mini Kit (Qiagen S.p.A., Milan, Italy), following the manufacturer’s protocol and the RNA templates were stored at –70°C until their use.

### Canine Pneumovirus Detection and Quantification

All RNA extracts were subjected to a previously-established RT-PCR assay for detection of CnPnV RNA [14], with minor modifications. Briefly, a one-step method was adopted using SuperScript™ One-Step RT-PCR for Long Templates (Invitrogen srl, Milan, Italy), according to the manufacturer’s instructions, and primers SH1F/SH187R that amplify a 208-bp of the small hydrophobic (SH) protein gene (Table 1). The following thermal protocol was used: reverse transcription at 50°C for 30 min, inactivation of Superscript II RT at 94°C for 2 min, 40 cycles of 94°C for 30 s, 54°C for 30 s, 68°C for 60 s, with a final extension at 68°C for 10 min. The PCR products were detected by electrophoresis through a 1.5% agarose gel and visualisation under UV light after ethidium bromide staining.

**Table 1:** Sequence, position and specificity of the oligonucleotides used in the study.

Assay	Reference	Primer/probe	Sequence 5′ to 3′	Polarity	Targetgene	Position^a^	Ampliconsize
Real-timeRT-PCR	This study	CnPnV-For	AAGATAAATTCTTCTATGAAAACAGAATGA	+	M2	8065–8094	110 bp
		CnPnV-Rev	CCCATCGTAAGTGAGGTTTCTATT	–		8151–8174	
		CnPnV-Pb	6-FAM-CTGCCTAAATACTATCCAGCCATACTGC-BHQ1	–		8100–8127	
Gel-basedRT-PCR	14	SH1F	ATGGATCCTAACATGACCTCACAC	+	SH	4104–4127	208 bp
		SH187R	GATTGGGATGAACCGTGCATTG	–		4290–4311	

^a^ Oligonucleotide positions are referred to the sequence of CnPnV strain dog/Ane4/USA/2008 (GenBank accession no. HQ734815).

In addition to the gel-based RT-PCR, a real-time RT-PCR assay based on the TaqMan technology was developed for the rapid detection and quantification of the CnPnV RNA in all clinical samples. Reactions were carried out using Platinum® Quantitative PCR SuperMix-UDG (Invitrogen srl) in a 50-µl mixture containing 25 µl of master mix, 300 nM of primers CnPnV-For and CnPnV-Rev, 200 nM of probe CnPnV-Pb (Table 1) and 10 µl of template RNA. Duplicates of log_10_ dilutions of standard RNA were analyzed simultaneously in order to obtain a standard curve for absolute quantification. The thermal profile consisted of incubation with UDG at 50°C for 2 min and activation of Platinum Taq DNA polymerase at 95°C for 2 min, followed by 45 cycles of denaturation at 95°C for 15 s, annealing at 48°C for 30 s and extension at 60°C for 30 s.

### Virus Isolation Attempts

CnPnV positive samples were inoculated into semiconfluent canine fibroma (A-72) cells, as previously described [14]. Inoculated cells were maintained in D-MEM supplemented with 5% FCS and monitored daily for the occurrence of cytopathic effect (CPE). After 6 days of incubation, the monolayers were tested for CnPnV antigen by an immunofluorescence (IF) assay using a monoclonal antibody targeting HRSV (Monosan®, Sanbio BV, Uden, The Netherlands). The cells were sub-cultured every 6–8 days for 5 consecutive passages.

### Sequence Analysis and Phylogeny

The RT-PCR products obtained with primer pair SH1F/SH187R were subjected to direct sequencing at the BaseClear B.V. (Leiden, The Netherlands). The sequences were manually edited and analyzed using the BioEdit software package [16] and the NCBI’s (htttp://www.ncbi.nlm.nih.gov) and EMBL’s (http://www.ebi.ac.uk) analysis tools.

In order to obtain new insights into the genetic diversity of CnPnV, the Italian prototype strain dog/Bari/100-12/ITA/2012 was submitted to RT-PCR amplification and subsequent sequence analysis of the full-length genome, using oligonucleotide retrieved from previous studies [13]–[14]. Additional RT-PCR assays with the enzyme mix SuperScript® II RT/Platinum® *Taq* Hi Fi (Invitrogen srl) and subsequent sequencing attempts were performed to close gaps between assembled contigs and to sequence unresolved genomic regions using primers designed on the alignment of the reference CnPnV strains with the closely related MPV. All the PCR amplicons were cloned and consensus sequences were elaborated using at least three clones per fragment. To determine the sequence of the 3′-leader and of the 5′-trailer ends (genome sense), viral RNA was reverse transcribed using NS2 negative-sense and L positive-sense primers, respectively, and the resulting cDNAs were amplified using a 5′ RACE kit (5′ RACE System for Rapid Amplification of cDNA Ends, Invitrogen srl), following the manufacturer’s instructions. Sequence analyses were conducted as for the SH fragment.

Phylogenetic and molecular evolutionary analyses were conducted using Mega4.1 Beta [17]. Phylogenetic trees based on the 8,598 nucleotide (nt) fragment available for extant CnPnVs and on the amino acid (aa) sequences of nucleocapsid (N) and fusion (F) proteins were elaborated using both parsimony and neighbor-joining methods, supplying a statistical support with bootstrapping over 1000 replicates. The following *Pneumovirus* reference strains were used for phylogeny (GenBank accession numbers are indicated in parentheses): CnPnV strains dog/Brne17/USA/2008 (GU247050) and dog/Ane4/USA/2008 (HQ734815); MPV strains 15 (AY729016) and J3666 (NC006579); HRSV strain B1 (NC_001781); BRSV strain ATue51908 (NC_001989). The distantly-related *Metapneumovirus* human metapneumovirus (HMPV) CAN97-83 (NC_004148) was used as outgroup.

### Nucleotide Sequence Accession Number

The full-length genome of the CnPnV strain dog/Bari/100-12/ITA/2012 was deposited in GenBank under accession number KF015281.

### Detection of Other Respiratory Pathogens

Respiratory specimens were submitted to molecular detection of other respiratory pathogens of dogs, such as canine parainfluenza virus (CPiV) [18], reoviruses [19], CAdVs [20], CDV [21], canine respiratory coronavirus (CRCoV) [22], canine pantropic coronavirus [23]–[24], canine minute virus [25], canid herpesvirus type 1 [26], canine influenza virus [27], canine hepacivirus [28], canine bocaviruses [9], *Bordetelella bronchiseptica* [29], *Streptococcus equi* subsp. *zooepidemicus* [30], *Mycoplasma cynos* and *Mycoplasma canis* [31].

Standardised procedures were carried out for in vitro isolation of other CIRD-associated bacteria. Samples were plated out on 5% sheep blood agar and cultured aerobically at 37°C for 24 h for the detection of aerobic pathogens. Bacteria were identified by standard biochemical procedures and analytical profile index (API, BioMérieux Italia S.p.A., Rome, Italy).

## Results

### Detection of CnPnV as Causative Agent of the Respiratory Outbreak

Both sampled dogs tested positive by RT-PCR targeting the SH gene of CnPnV. By sequence analysis of the PCR product, a 100% nucleotide (nt) identity was found between the two detected CnPnV strains. By means of real-time RT-PCR, the dogs were confirmed to be infected by CnPnV, displaying discrete viral titers, which were 2.06×10^5^ (dog 100/12) and 1.59×10^4^ (dog 101/12) RNA copies µl^−1^ of template.

No other respiratory pathogens were detected in the analyzed samples. All attempts to isolate either CnPnV strains were unsuccessful, as shown by the absence of CPE and by negative IF testing.

### Full-genomic Characterisation of the Italian CnPnV Prototype Strain

**Figure 1:**
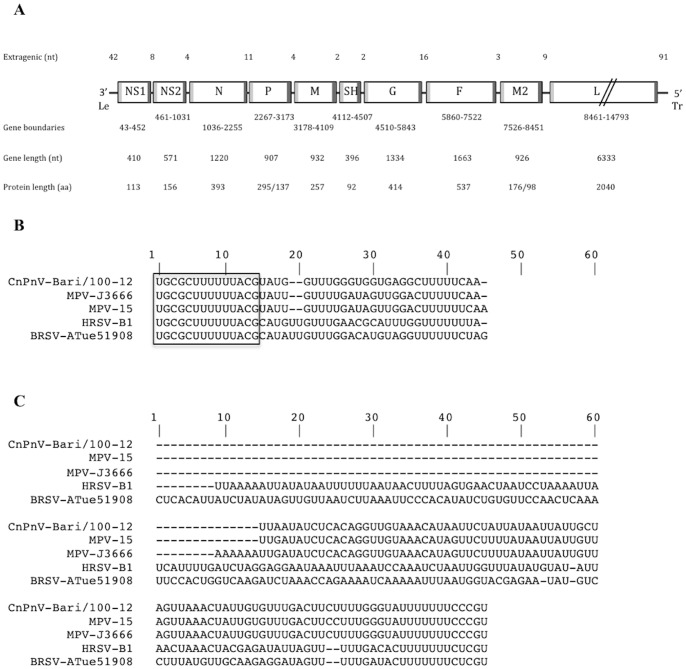
Genomic organisation of CnPnV strain Bari/100-12. **A.** A map of the viral genome is shown (not to scale), drawn 3′ to 5′ as negative-sense RNA such that the direction of transcription is from left to right. Genes are shown as rectangle, with the shaded bars at each end representing the gene-start (lighter shading) and gene-end (darker shading) transcription signals. The horizontal line represents the extargenic regions, specifically the 3′-leader (Le), 5′-trailer (Tr) and intergenic regions. Nucleotide (nt) length for extragenic sequences are indicated above the map; below the gene map are the gen coordinates, the nt lengths of the mRNAs and the amino acid (aa) lengths of the predicted proteins. P and M2 genes are predicted to encode each for two different proteins. **B, C.** CnPnV/Bari/100-12 leader (**B**) and trailer (**C**) regions aligned with the analogous sequences of other pneumoviruses. All sequences are shown in the negative genome sense from 3′ to 5′. The conserved 14 nt stretch at the 3′ end of the leader region is boxed.

The virus detected in one dog (dog/Bari/100-12/ITA/2012) was assumed as prototype of the CnPnV strains circulating in the shelter and submitted to full-genomic sequence analysis. This showed that the genome of strain dog/Bari/100-12/ITA/2012 (Bari/100-12) was 14,884 nucleotides (nt) in length and displayed the same organization as MPV with 10 genes that encode for 12 putative proteins. The same genomic organization was found as in MPV with the gene order 3′-NS1-NS2-N-P-M-SH-G-F-M2-L-5′ and with the coding regions being flanked by leader and trailer regions at the 3′ and 5′ ends (genome sense), respectively (Fig. 1A). The genome size was 1 and 3 nt shorter than that of MPV isolates J3666 and 15, respectively, whereas no comparison was possible with extant CnPnVs whose full-length genomes are not available [14].

The full-length genome of the Italian CnPnV strain displayed the highest nt identity (95.7–95.8%) to MPV, whereas the genetic relatedness to HRSV and BRSV was less than 50% (Table 2). When the analysis was restricted to the 8,600 and 8,598 nt available for reference CnPnV strains Brne17 and Ane4, respectively, that span from the very 3′ end of the L gene to the 5′end of the leader region (genome sense), an overall sequence identity of 96.5–96.6% was found against the canine strains. This identity was only slightly higher than that displayed against MPVs (94.8–95%, Table 2).

**Table 2:** Nucleotide identities (%) of CnPnV dog/Bari/100-12/ITA/2012 with reference pneumoviruses in different genomic regions.

Pneumovirus strain(GenBank accession number)	Leaderregion	NS1	NS2	N	P	M	SH	G	F	M2	L	Trailerregion	Full-lengthgenome	8598-ntfragment
**CnPnV/Brne17 (GU247050)**	NA	94.7	94.7	97.2	97.4	96.8	95.5	96.4	97.4	96.4	N.A.	NA	NA	96.5
**CnPnV/Ane4 (HQ734815)**	NA	94.7	94.7	97.1	97.4	96.9	95.5	96.6	97.4	96.4	N.A.	NA	NA	96.6
**MPV/strain 15 (AY729016)**	78.6	94.2	94.2	95.9	94.8	95.3	89.4	94.1	96.8	95.1	96.7	94.5	95.7	94.8
**MPV/J3666 (NC_006579)**	83.3	94.2	94.2	95.8	95.6	95.4	90	94.4	96.9	95.1	96.6	95.6	95.8	95
**HRSV/B1 (NC_001803)**	68.2	39.6	39.9	61.4	45.8	49.5	44.7	36.8	49.1	44.6	54	52.7	48.7	51.1
**BRSV**/**ATue51908 (NC_001989)**	72.7	36.7	38.6	60	44.8	50.9	32.5	35.3	50.5	45	54	47.3	48.6	51.5

NA, sequence not available.

In the negative genome sense, the leader region consisted of a 42 nt U-rich sequence as for that of strain MPV/J3666, whereas it was 1, 2 and 3 nt shorter than the same sequence of MPV/15, HRSV and BRSV (Fig. 1B). The nt identity to other members of the genus *Pnuemovirus* ranged from 68.2% (HRSV) to 83.3% (MPV/J3666). Only the 5′-end 25 nt had been sequenced for the reference CnPnV strains showing a 100% identity to the same stretch of the leader region of strain Bari/100-12. The 14 nt at the 3′ end were conserved among pneumoviruses.

The A-rich trailer sequence at the 5′ end (genome sense) was 91 nt long as that of MPV strains 15 and J3666, whereas the corresponding sequences of HRSV and BRSV were 154 and 161 nt long (Fig. 1C). The nt identity was very high to MPV isolates (94.5–95.6%), being markedly lower to HRSV (52.7%) and BRSV (47.3%). No comparison was possible with the analogous sequences of CnPnV strains Brne17 and Ane4 (that had not been determined), whereas a conserved 5′-end stretch of 12 nt was found among all pneumoviruses analysed with the exception of the fourth position where HRSV and BRSV exhibited the substitution G to A (Fig. 1C).

Non-translated regions were found to be located between the protein coding regions, including gene start (GS) and gene end (GE) sequences that define the transcriptional boundaries on the negative strand template and short intergenic regions (IGR) lying between the GE of one gene and the GS of the following coding region (data not shown).

Nucleotide and amino acid identities of the Italian CnPnV to other pneumoviruses in the different genomic regions are shown in Table 2 and Table 3, respectively, whereas Table 4 shows the aa substitutions encountered in the encoded proteins in comparison to extant CnPnVs and MPVs. Strain Bari/100-12 displayed intact genes for non-structural proteins NS1 (410 nt) and NS2 (571 nt) with respect to extant CnPnVs and MPV/15, whereas the same genes were shorter for MPV/J3666 (406 and 566 nt, respectively). HRSV and BRSV had longer NS1 (532 and 527 nt, respectively) and shorter NS2 sequences (502 and 494 nt, respectively). In these genomic regions gene strain Bari/100-12 was closely related to other CnPnVs (94.7% of nt identity), as well as to MPV (94.2% of nt identity). The encoded proteins NS1 and NS2 had the same length (113 and 156 aa, respectively) in all CnPnVs and MPVs, with the Bari/100-12 NS1 and NS2 products being more closely related to the latter viruses (99.1% and 95.5% of aa identity, respectively). While the former protein showed only 1 aa variation in comparison to extant CnPnVs, 10 substitutions were encountered in the NS2 polypeptide.

**Table 3:** Amino acid identities (%) of CnPnV dog/Bari/100-12/ITA/2012 with reference pneumoviruses in different genomic regions.

Pneumovirus strain(GenBank accession number)	NS1	NS2	N	P	M	SH	G	F	M2-1	M2-2	L
**CnPnV/Brne17 (GU247050)**	99.1	93.6	98.5	99.7	98.1	95.7	93.4	98.3	98.9	99	NA
**CnPnV/Ane4 (HQ734815)**	99.1	93.6	98.5	99.7	98.5	95.7	97.1	98.3	98.9	99	NA
**MPV/strain 15 (AY729016)**	92.9	95.5	97.7	95.3	98.1	92.4	92.2	98	97.7	96.9	98.5
**MPV/J3666 (NC_006579)**	92.9	95.5	97.5	96.9	98.1	97.8	91.9	98.3	97.7	96.9	98.5
**HRSV/B1 (NC_001803)**	14.8	16.7	59	33.1	41	15.7	8.9	40.4	42.1	13.3	49.9
**BRSV**/**ATue51908 (NC_001989)**	11.9	18.1	59.5	36.2	41	16.1	13.2	41.6	42.1	14.3	49.1

NA, sequence not available.

**Table 4:** Substitutions in the proteins of CnPnV/Bari/100-12 compared with extant canine and murine pneumoviruses.

Protein name	Nt position^a^	Protein residue^a^	CnPnV/Bari/100–12	CnPnV/Brne17	CnPnV/Ane4	MPV/15	MPV/J3666
NS1	187	36	D	G	G	G	G
NS2	507	12	V	I	I	I	I
	555	28	G	S	S	G	G
	570	33	V	M	M	V	V
	672	67	W	R	R	W	W
	774	101	A	S	S	S	S
	816	115	E	K	K	E	E
	832	120	V	E	E	V	V
	876	135	V	I	I	I	I
	899	142	P	L	L	P	P
	939	156	N	D	D	D	D
N	1533	156	Y	C	C	C	C
	1547	161	V	L	L	L	L
	1553/1554	163	G	V	V	I	I
	1730	222	I	V	V	V	V
	2091	342	R	K	K	R	R
	2214	383	N	S	S	N	N
P	2990/2992	239	I	V	V	I	I
M	3321	45	K	R	R	K	K
	3496	103	D	E	E	D	D
	3557	124	D	N	N	N	N
	3720	178	Q	R	Q	Q	Q
SH	4257	46	V	I	I	I	I
	4300	60	V	A	A	V	V
	4350	77	V	I	I	V	V
	4362	81	G	S	S	G	G
G	4544	3	P^b^	T	T	/	/
	4548/4549	4	A	V	V	/	/
	4613	26	S^b^	G	G	S	S
	4623	29	T	I	I	I	I
	4670	45	A	T	T	T	T
	4794	86	M^b^	T	T	T	T
	4851	105	P^b^	L	L	L	L
	4883	116	F^b^	L	L	F	F
	4935	133	A	/	V	V	V
	5036	167	I^b^	/	L	I	I
	5325	263	A^b^	/	V	A	A
	5703	389	D^b^	/	G	D	D
F	6110	81	S	N	N	S	S
	6364	166	E	K	K	K	K
	7202	445	S	N	N	S	S
	7285	473	N	D	D	D	D
	7316	483	E	V	V	E	E
	7331	488	N	T	T	N	N
	7388	507	A	V	V	A	A
	7417/7418	517	L	S	S	L	L
	7439	524	K	R	R	K	K
M2-1	7750	57	S	T	T	S	S
	7952/7943	121	T	V	V	T	T

^a^ Nt positions and protein residues are referred to the sequences of CnPnV strain Bari/100-12 (GenBank accession number KF015281).

^b^ Mutations detected in the G protein of recently analysed carnivore pneumoviruses [32].

/, Residue not present.

The N protein gene was 1,220 nt long, as for extant CnPnVs, which is 1 and 5 nt longer than the same region of MPVs 15 and J3666, respectively. The nt identity in this region ranged from 60% for BRSV to 97.2% for CnPnV/Brne17. The relatedness to other CnPnVs (98.5% of aa identity) was confirmed in the putative N protein (393 aa), which displayed 6 substitutions among canine viruses.

The phosphoprotein (P) gene had the same length (907 nt) as for reference CnPnVs, which was the longest among analysed pneumoviruses. The nt identity was 97.4% against other CnPnVs and 94.8–95.6% against MPVs. Two different products, 295 and 137 aa long respectively, are encoded by this gene. The major polypeptide of strain Bari/100-12 displays the best aa identity (99.7%) against other canine isolates and only 1 aa change over other CnPnVs.

The matrix (M) protein gene was 932 nt long as for CnPnVs Brne17 and Ane4, but it was 1 and 5 nt longer than in MPVs 15 and J3666, respectively. The genic relatedness against other canine and murine isolates was 96.8–96.9% and 95.3–95.4% of nt identity, respectively. The putative M protein was 257 aa long and exhibited approximately the same aa identity (98.1–98.5%) with canine and murine strains, with 4 mutations with respect to American strains.

The small hydrophobic (SH) protein gene displayed the same length as CnPnV/Ane4 and MPVs, whereas in strain Brne17 this sequence was 1 nt shorter. The genetic distance to MPVs was higher in the SH gene than in other regions, with only 89.4–90% nt identity in comparison to an identity of 95.5% showed against extant canine strains. However, in the encoded protein (92 aa long), while the aa identity to strain MPV/J3666 was again high (97.8%), MPV/15 showed a lower relatedness with 92.4% of aa identity. Four changes were detected among the SH products of the different canine viruses.

The attachment (G) protein gene had the same length (1,334 nt) in all CnPnV strains, which was 1 and 5 nt longer that that of murine strains 15 and J3666, respectively. The analogous gene of HRSV and BRSV was 412 and 494 nt shorter, respectively. The genetic relatedness to other canine isolates (96.4–96.6% nt identity) was slightly higher with respect to MPVs (94.1–94.4%). G sequences are available for additional 10 carnivore pneumoviruses, two of which are of feline origin [32]. When these sequences were included in the analyses, the overall nt identity to Italian CnPnVs ranged from 91.6% to 98.2%.

The encoded G protein of canine and murine pneumoviruses had different length ranging from 121 aa (strain CnPnV/Brne17) to 414 aa (strains CnPnV Bari/100-12 and Ane4). Thus, the aa identities to strain CnPnV-Bari/100-12 ranged from 91.9% (MPV/J3666) to 97.1% (CnPnV/Ane4). However, when recent CnPnVs were included, the aa identities varied from 89.6% to 95.9%. Apart from the 293-aa deletion observed in CnPnV/Brne17, 12 mutations were evident in this product, 8 of which were also present in the G sequences of carnivore pneumovirus recently analysed (Table 4).

An opposite situation was found in the F protein gene, were HRSV and BRSV displayed sequences that were 239–240 nt longer than those of canine pneumoviruses (1,663 nt) and 240–246 nt longer than those of murine strains (1,657–1,662 nt). Also in this region the nt identities to other CnPnVs were slightly higher than to MPVs (97.4% against 96.8–96.9%), whereas in the encoded protein (537 aa) the aa identities were approximately equal for canine and murine strains (98–98.3%). The F protein displayed 9 aa changes among CnPnVs.

The MPV M2 gene had been suggested to be 875 nt long [33], whereas Renshaw et al. [14] reported a 926-nt M2 sequence for CnPnVs based on the presence of the GS sequence upstream the location previously detected for MPV [34]. Consequently, the M2 gene length was re-determined as 928 and 921 nt for MPV isolates 15 and J3666, respectively. In strain Bari/100-12, this gene was 926 nt long and shared a 96.4% and 95.1% nt identity with other CnPnVs and MPVs, respectively. Interestingly, the genetic relatedness was higher to HPMV (46.2% nt identity) than to HRSV/BRSV (44.6–45%). The *Pneumovirus* M2 gene is usually translated into two proteins, M2-1 and M2-1, from alternative reading frames. In both proteins that were 176 and 98 aa in length, respectively, the Italian CnPnV was more closely related to extant canine viruses than to MPVs (aa identities of 98.9% and 99% in the M2-1 and M2-2 products, respectively). The M2-1 protein showed 2 aa substitutions, whereas the M2-2 product was conserved among CnPnVs.

Comparison of the Bari/100-12 large polymerase (L) gene with that of other canine strains was not possible since those sequences have not been determined for CnPnVs. The L gene of CnPnV-Bari/100-12 was 6,333 long showing similar length in comparison with MPV isolates 15 (6,332 nt) and J3666 (6,326 nt). In this region, the nt identity against murine strains was 96.6–96.7%. Interestingly, HMPV was more closely related to strain Bari/100-12 (56.3% nt identity) than were HRSV and BRSV (both displaying 54% nt identity). However, when the aa sequence was analysed, the genetic relatedness to HRSV/BRSV (49.1–49.9% aa identity) was higher than that with HPMV (48.6% aa identity).

### Phylogeny

**Figure 2:**
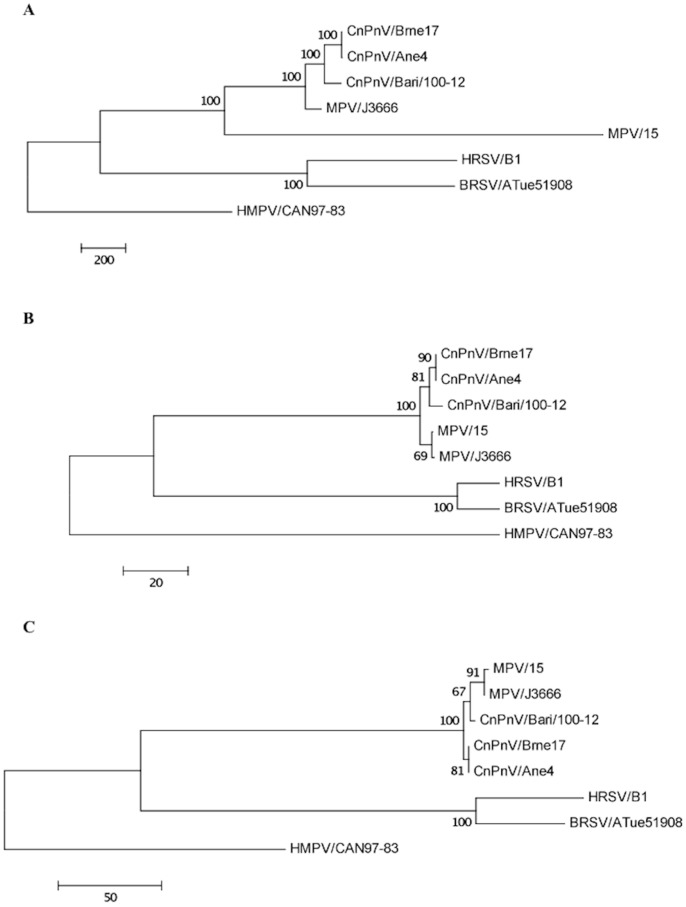
Phylogenetic analysis of CnPnV strain Bari/100-12 and other members of the genus *Pneumovirus*. Maximum parsimony trees are based on the 8,598-nt fragment spanning from the very 3′ end of the L gene to the 5′end of the leader region (**A**), nucleocapsid (**B**) and fusion (**C**) proteins. For phylogenetic tree construction, the reference strains and GenBank accession numbers are as reported in Tables 2 and 3. The distantly-related *Metapneumovirus* human metapneumovirus (HMPV) CAN97-83 (NC_004148) was used as outgroup. A statistical support was provided by bootstrapping over 1,000 replicates. The scale bars indicate the estimated numbers of nucleotide or amino acid substitutions.

In order to include in the analysis the nt sequences available for extant CnPnVs, phylogeny was first constructed on the 8,598-nt fragment spanning from the very 3′ end of the L gene to the 5′end of the leader region (genome sense) [14]. In the maximum parsimony tree elaborated using these sequences, strain CnPnV/Bari/100-12 clustered with other CnPnV isolates and with the murine strain J3666, whereas MPV/15 was more distantly related (Fig. 2A). The Italian CnPnV was tightly intermingled with extant canine isolates also in the tree constructed on the N protein (Fig. 2B), whereas that obtained from the F protein revealed an unexpected subclustering with murine strains (Fig. 2C). The same tree topologies were obtained through phylogenetic analysis using the neighbor-joining method (data not shown).

## Discussion

In recent years, novel viruses have been recognized as causative agents of CIRD, including influenza viruses [5], hepaciviruses [28], and bocaviruses [9]. CnPnV was first detected in 2 animal shelters with CIRD in the northeastern United States during 2008–2009 [13]. Although CnPnV was discovered at least 4 years ago, only two thirds of the viral genome have been analysed so far [14].

In this paper, the identification of CnPnV in Italy is reported together with a further evidence of its association to CIRD. The full-length genome of the Italian prototype strain was determined, which confirmed the close genetic relatedness of strain Bari/100-12 to the American isolates and of CnPnV to MPV. Although CnPnV has been very recently detected in UK [35], no sequences are yet available from those isolates, thus preventing any sequence comparison among European CnPnVs.

Although MPV was first identified in the 1940s, to date most reports of virus detection are from laboratory mouse and rat colonies. A 10-year survey in French laboratory rodents showed that 13.9% and 15.9% of the tested rat and mouse colonies, respectively, had antibodies against MPV [36]. Serosurveys conducted in wild rodents in UK gave inconsistent results, with antibodies being detected in certain species of wild voles and mice [37] and in wild grey squirrels (*Sciurus carolinensis*) [38], but not in wild house mice (*Mus musculus*) [39]. In Italy there are no recent data about the MPV circulation in laboratory or wild rodents. However, if the mouse virus circulates in the field, there are several chances for its species jump to the canine host. In this scenario, kenneled dogs should be highly exposed to the cross-species transmission, considering that shelters usually attract large populations of rodents and pest control measures are not carried out on a regular basis. Apart from kenneled dogs, hunting dogs could be also exposed to MPV infection through occasional contacts with wild rodents during hunting activities. Introduction of MPV-like viruses in Italy may have occurred through migration of infected wild rodents or importation of infected dogs from other countries.

In addition, with the limited data available so far, whether the murine viruses are ancestors of the canine strains or the two groups of viruses co-evolved independently from a common ancestor could not be assessed. Further studies providing additional canine and murine viral sequences are needed in order to date back the emergence of CnPnV.

Despite the presence of discrete viral titres in the clinical samples, as calculated by a newly established real-time RT-PCR assay, attempts to isolate in vitro the Italian CnPnV were unsuccessful. This finding could be explained with virus inactivation during sample transportation or storage or, alternatively, with poor sensitivity of the canine cells employed for virus isolation. In fact, even if A-72 cells have been reported to be highly permissive for CnPnV replication [14], virus adaptation to the in-vitro growth may vary based on different clones of the same cell line due to different levels of expression of cell receptors.

Currently, the pathogenic potential of CnPnV is not completely known. However, recent reports of natural infections in dogs [13] and experimental infections in mice [15], [32] have suggested its potential role in the occurrence of CIRD. Additional evidence has been very recently obtained during an epidemiological survey carried out in the United Kingdom [35]. In that study, CnPnV was detected in tracheal tissues of 29/205 kenneled dogs, most of which had suffered from mild to moderate respiratory disease. The role of CnPnV in the canine respiratory disease is also corroborated by the findings reported in the present paper where CnPnV strain Bari/100-12 was associated to acute respiratory disease in a shelter. Although the laboratory investigations failed to detect additional agents of CIRD, other, less common causes cannot be definitively ruled out.

Kennels and shelters are likely to be mostly exposed since the poor hygienic conditions and the overcrowding may facilitate the widespread circulation and increased virulence of the multiple pathogens responsible for the respiratory disease [1]. Further studies will elucidate the pathogenicity and epidemiology of this novel pneumovirus, thus addressing the eventual need for specific vaccines.

## References

[1] Buonavoglia C, Martella V (2007) Canine respiratory viruses. Vet Res 38: 355–373. 17296161 10.1051/vetres:2006058

[2] Decaro N, Martella V, Buonavoglia C (2008) Canine adenoviruses and herpesvirus. Vet Clin North Am Small Anim Pract 38: 799–814. 18501279 10.1016/j.cvsm.2008.02.006 PMC7114865

[3] Martella V, Elia G, Buonavoglia C (2008) Canine distemper virus. Vet Clin North Am Small Anim Pract 38: 787–797. 18501278 10.1016/j.cvsm.2008.02.007

[4] Ellis JA, Krakowka GS (2012) A review of canine parainfluenza virus infection in dogs. J Am Vet Med Assoc 240: 273–284. 22256842 10.2460/javma.240.3.273

[5] Dubovi EJ (2010) Canine influenza. Vet Clin North Am Small Anim Pract 40: 1063–1071. 20933136 10.1016/j.cvsm.2010.07.005 PMC7132494

[6] Erles K, Toomey C, Brooks HW, Brownlie J (2003) Detection of a group 2 coronavirus in dogs with canine infectious respiratory disease. Virology 310: 216–223. 12781709 10.1016/S0042-6822(03)00160-0 PMC7126160

[7] Decaro N, Buonavoglia C (2008) An update on canine coronaviruses: Viral evolution and pathobiology. Vet Microbiol 132: 221–234. 18635322 10.1016/j.vetmic.2008.06.007 PMC7117484

[8] Decaro N, Buonavoglia C (2011) Canine coronavirus: not only an enteric pathogen. Vet Clin North Am Small Anim Pract 38: 799–814. 10.1016/j.cvsm.2011.07.005 PMC7114679 22041207

[9] Kapoor A, Mehta N, Dubovi EJ, Simmonds P, Govindasamy L, et al (2012) Characterization of novel canine bocaviruses and their association with respiratory disease. J Gen Virol 93: 341–346. 22031527 10.1099/vir.0.036624-0 PMC3352345

[10] Kapoor A, Simmonds P, Gerold G, Qaisar N, Jain K, et al (2011) Characterization of a canine homolog of hepatitis C virus. Proc Natl Acad Sci USA 108: 11608–11613. 21610165 10.1073/pnas.1101794108 PMC3136326

[11] Fauquet CM, Mayo MA, Maniloff J, Desselberger U, Ball LA (2005) Virus Taxonomy. Eighth Report of the International Committee on Taxonomy of Viruses. London: Elsevier/Academic Press.

[12] Zenner L, Regnault JP (2000) Ten-year long monitoring of laboratory mouse and rat colonies in French facilities: a retrospective study. Lab Anim 34: 76–83. 10759370 10.1258/002367700780577957

[13] Renshaw RW, Zylich NC, Laverack MA, Glaser AL, Dubovi EJ (2010) Pneumovirus in dogs with acute respiratory disease. Emerg Infect Dis 16: 993–995. 20507755 10.3201/eid1606.091778 PMC3086219

[14] Renshaw R, Laverack M, Zylich N, Glaser A, Dubovi E (2011) Genomic analysis of a pneumovirus isolated from dogs with acute respiratory disease. Vet Microbiol 150: 88–95. 21324612 10.1016/j.vetmic.2011.01.013

[15] Percopo CM, Dubovi EJ, Renshaw RW, Dyer KD, Domachowske JB, et al (2011) Canine pneumovirus replicates in mouse lung tissue and elicits inflammatory pathology. Virology 416: 26–31. 21600624 10.1016/j.virol.2011.04.010 PMC3112256

[16] Hall TA (1999) BioEdit: a user-friendly biological sequence alignment and analysis program for Windows 95/98/NT. Nucl Acids Symp Ser 41: 95–98.

[17] Tamura K, Dudley J, Nei M, Kumar S (2007) MEGA4: Molecular Evolutionary Genetics Analysis (MEGA) software version 4.0. Mol Biol Evol 24: 1596–1599. 17488738 10.1093/molbev/msm092

[18] Erles K, Dubovi EJ, Brooks HW, Brownlie J (2004) Longitudinal study of viruses associated with canine infectious respiratory disease. J Clin Microbiol 42: 4524–4529. 15472304 10.1128/JCM.42.10.4524-4529.2004 PMC522361

[19] Leary TP, Erker JC, Chalmers ML, Cruz AT, Wetzel JD, et al (2002) Detection of mammalian reovirus RNA by using reverse transcription-PCR: sequence diversity within the λ3-encoding L1 gene. J Clin Microbiol 40: 1368–1375. 11923358 10.1128/JCM.40.4.1368-1375.2002 PMC140344

[20] Hu RL, Huang G, Qiu W, Zhong ZH, Xia XZ, et al (2001) Detection and differentiation of CAV-1 and CAV-2 by polymerase chain reaction. Vet Res Commun 25: 77–84. 11214675 10.1023/a:1006417203856

[21] Elia G, Decaro N, Martella V, Cirone F, Lucente MS, et al (2006) Detection of canine distemper virus in dogs by real-time RT-PCR. J Virol Methods 136: 171–176. 16750863 10.1016/j.jviromet.2006.05.004

[22] Decaro N, Elia G, Campolo M, Desario C, Mari V, et al (2008) Detection of bovine coronavirus using a TaqMan-based real-time RT-PCR assay. J Virol Methods. 151: 167–171. 10.1016/j.jviromet.2008.05.016 PMC7112840 18579223

[23] Decaro N, Pratelli A, Campolo M, Elia G, Martella V, et al (2004) Quantitation of canine coronavirus RNA in the faeces of dogs by TaqMan RT-PCR. J Virol Methods. 119: 145–150. 10.1016/j.jviromet.2004.03.012 PMC7119844 15158596

[24] Decaro N, Martella V, Ricci D, Elia G, Desario C, et al (2005) Genotype-specific fluorogenic RT-PCR assays for the detection and quantitation of canine coronavirus type I and type II RNA in faecal samples of dogs. J Virol Methods 130: 72–78. 16024100 10.1016/j.jviromet.2005.06.005 PMC7112928

[25] Decaro N, Altamura M, Pratelli A, Pepe M, Tinelli A, et al (2002) Evaluation of the innate immune response in pups during canine parvovirus type 1 infection. New Microbiol 25: 291–298. 12173770

[26] Decaro N, Amorisco F, Desario C, Lorusso E, Camero M, et al (2010) Development and validation of a real-time PCR assay for specific and sensitive detection of canid herpesvirus 1. J Virol Methods 169: 176–180. 20674611 10.1016/j.jviromet.2010.07.021 PMC7112867

[27] Di Trani L, Bedini B, Donatelli I, Campitelli L, Chiappini B, et al (2006) A sensitive one-step real-time PCR for detection of avian influenza viruses using a MGB probe and an internal positive control. BMC Infect Dis 6: 87. 16725022 10.1186/1471-2334-6-87 PMC1524785

[28] Bukh J (2011) Hepatitis C homolog in dogs with respiratory illness. Proc Natl Acad Sci USA 108: 12563–12564. 21768355 10.1073/pnas.1107612108 PMC3150949

[29] Zhao Z, Wang C, Xue Y, Tang X, Wu B, et al (2011) The occurrence of *Bordetella bronchiseptica* in pigs with clinical respiratory disease. Vet J 188: 337–340. 20598597 10.1016/j.tvjl.2010.05.022

[30] Priestnall SL, Erles K, Brooks HW, Cardwell JM, Waller AS, et al (2010) Characterization of pneumonia due to *Streptococcus equi* subsp. *zooepidemicus* in dogs. Clin Vaccine Immunol 17: 1790–1796. 20861329 10.1128/CVI.00188-10 PMC2976090

[31] Chalker VJ, Owen WM, Paterson C, Barker E, Brooks H, et al (2004) Mycoplasmas associated with canine infectious respiratory disease. Microbiology 150: 3491–3497. 15470126 10.1099/mic.0.26848-0

[32] Glineur SF, Renshaw RW, Percopo CM, Dyer KD, Dubovi EJ, et al (2013) Novel pneumoviruses (PnVs): Evolution and inflammatory pathology. Virology 443: 257–264. 23763766 10.1016/j.virol.2013.05.011 PMC3722285

[33] Krempl CD, Lamirande EW, Collins PL (2005) Complete sequence of the RNA genome of pneumonia virus of mice (PVM). Virus Genes 30: 237–249. 15744580 10.1007/s11262-004-5631-4

[34] Dibben O, Easton AJ (2007) Mutational analysis of the gene start sequences of pneumonia virus of mice. Virus Res 130: 303–309. 17658649 10.1016/j.virusres.2007.06.009

[35] Mitchell JA, Cardwell JM, Renshaw RW, Dubovi EJ, Brownlie J (2013) The detection of canine pneumovirus in dogs with canine infectious respiratory disease. J Clin Microbiol doi:10.1128/JCM.02312–13. 10.1128/JCM.02312-13 PMC3838075 24088858

[36] Zenner L, Regnault JP (2000) Ten-year long monitoring of laboratory mouse and rat colonies in French facilities: A retrospective study. Lab Anim 34: 76–83. 10759370 10.1258/002367700780577957

[37] Kaplan C, Healing TD, Evans N, Healing L, Prior A (1980) Evidence of infection by viruses in small British field rodents. J Hyg (Lond) 84: 285–294. 6244344 10.1017/s0022172400026784 PMC2133884

[38] Greenwood AG, Sanchez S (2002) Serological evidence of murine pathogens in wild grey squirrels (*Sciurus carolinensis*) in North Wales. Vet Rec 150: 543–546. 12019534 10.1136/vr.150.17.543

[39] Becker SD, Bennett M, Stewart JP, Hurst JL (2007) Serological survey of virus infection among wild house mice (*Mus domesticus*) in the UK. Lab Anim 41: 229–238. 17430622 10.1258/002367707780378203

